# A User-Configurable Headstage for Multimodality Neuromonitoring in Freely Moving Rats

**DOI:** 10.3389/fnins.2016.00382

**Published:** 2016-08-19

**Authors:** Kanokwan Limnuson, Raj K. Narayan, Amrit Chiluwal, Eugene V. Golanov, Chad E. Bouton, Chunyan Li

**Affiliations:** ^1^Cushing Neuromonitoring Laboratory, The Feinstein Institute for Medical ResearchManhasset, NY, USA; ^2^Department of Neurosurgery, Hofstra Northwell School of MedicineHempstead, NY, USA; ^3^Center for Bioelectronic Medicine, The Feinstein Institute for Medical ResearchManhasset, NY, USA

**Keywords:** multimodal brain monitoring, headstage, ECoG, electrophysiology, cortical temperature, brain oxygen tension, cerebral blood flow, freely moving rat

## Abstract

Multimodal monitoring of brain activity, physiology, and neurochemistry is an important approach to gain insight into brain function, modulation, and pathology. With recent progress in micro- and nanotechnology, micro-nano-implants have become important catalysts in advancing brain research. However, to date, only a limited number of brain parameters have been measured simultaneously in awake animals in spite of significant recent progress in sensor technology. Here we have provided a cost and time effective approach to designing a headstage to conduct a multimodality brain monitoring in freely moving animals. To demonstrate this method, we have designed a user-configurable headstage for our micromachined multimodal neural probe. The headstage can reliably record direct-current electrocorticography (DC-ECoG), brain oxygen tension (PbrO_2_), cortical temperature, and regional cerebral blood flow (rCBF) simultaneously without significant signal crosstalk or movement artifacts for 72 h. Even in a noisy environment, it can record low-level neural signals with high quality. Moreover, it can easily interface with signal conditioning circuits that have high power consumption and are difficult to miniaturize. To the best of our knowledge, this is the first time where multiple physiological, biochemical, and electrophysiological cerebral variables have been simultaneously recorded from freely moving rats. We anticipate that the developed system will aid in gaining further insight into not only normal cerebral functioning but also pathophysiology of conditions such as epilepsy, stroke, and traumatic brain injury.

## Introduction

Multimodal brain monitoring aids in the understanding of brain physiology by tracking multiple parameters and by providing a comprehensive assessment (Vespa, 2005; Tisdall and Smith, 2007; Wartenberg et al., 2007; Oddo et al., 2012; Bouzat et al., 2015; Frontera et al., 2015). Brain function has been extensively investigated through its electrophysiological, biochemical, and physiological activities (He et al., 2008; Ge et al., 2012; Sanacora et al., 2012; Kiyatkin et al., 2013; Gonzalez et al., 2015; Prasad and Bondy, 2015). With recent progress in micro- and nanotechnology, micro-nano-implants are playing an increasingly crucial role in advancing brain research (Lee et al., 2009; Du et al., 2011; Dimitriadis et al., 2014; Xiang et al., 2014; Park et al., 2015; Son et al., 2015; Wang et al., 2015; Wei et al., 2015; Yang et al., 2015). Moreover, the recording electronics with advanced application specific integrated circuits (ASIC) can be interconnected with neural probes in different ways to form self-contained devices. However, the development of the neural probes and their interface circuits are mainly focused on a single modality, i.e., the same type of sensor (Yang et al., 2015; Scholvin et al., 2016). In order to realize the full potential of multimodality monitoring and better understand pathophysiologic relationships, multiple brain parameters need to be monitored. Unfortunately, there are no ready-to-use neural probes and/or interface circuits that are specifically designed for multimodality monitoring in animal research. In the absence of such probes and interface circuits, multiple electrodes and their individual interface circuits are needed to conduct multimodality monitoring (Ko, 2013). This has made multimodality neuromonitoring in freely moving animals extremely difficult.

Currently there are three general types of brain monitoring systems for rodents—a system for anesthetized animals; for awake animals, there is a tethered monitoring system and a wireless system. Under anesthesia, the measurement becomes simple since there is no artifact from animal movement (Suzuki et al., 2012). In addition, there's no critical requirement for the interfacing circuits such as, weight, dimensions, power consumption, etc. However, the anesthetic agent can affect the study outcome (Karmarkar et al., 2010). Another disadvantage of this system is that it is limited to short-term monitoring. In the tethered system, the animal is connected to the monitoring system through wires/cables. While this system allows brain monitoring in awake animals (Miao et al., 2011; Kerr and Nimmerjahn, 2012; Kealy et al., 2013; Dimitriadis et al., 2014; Urban et al., 2015; Wang et al., 2015), it imposes physical and behavioral restrictions on them. Moreover, the movements of suspended cables as well as the mechanical stress on the headstage connectors are significant sources of noise. A commutator is necessary to minimize tangling of connecting cables, but since this is in the direct signal path, it can also introduce undesirable noise. Alternatively, the use of a wireless system enables low-noise recording in a freely-moving animal. There have been many wireless headstages designed in an attempt to solve the problems associated with tethered systems with varying levels of success (DeBow and Colbourne, 2003; Harrison et al., 2007; Lapray et al., 2008; Ye et al., 2008; Silasi et al., 2009; Fan et al., 2011; Szuts et al., 2011; Russell et al., 2012; Chang and Chiou, 2013; Pinnell et al., 2015). An ideal wireless system must be small enough to sit on a small animal's head and be barely noticeable so that it does not impose significant burden on the animal. It should also use ultra-low power to provide maximum battery life for long experiments (Ball et al., 2014), or have the ability for wireless powering (Chang and Chiou, 2013).

To conduct multimodal brain monitoring, multiple customized interface circuits or commercialized rack-mounted electronics are normally required to measure variables with different signal levels and operation principles (Manor et al., 2008; Li et al., 2016a). The main advantage of this design is the flexible configurations to ensure best fit with the application at hand or experimental design. However, this system usually requires multiple cables from different interface electronics connecting directly to the neural probes, and therefore, is not suitable for conducting experiments in freely moving animals. Another way to achieve multimodal monitoring is by using commercial off-the-shelf (COTS) integrated circuits (ICs) to build a small headstage system for the freely moving rodents (Ball et al., 2014). The advantage of this approach is that the system can be quickly built from a combination of commercially available devices. It can have high configurability in terms of modifying the functionality when it incorporates other devices e.g., microprocessor. However, the amount of circuit components and processing systems that can be implemented in a single headstage is limited due to the limited dimension and weight that can be attached to a freely moving rodent. As the number of monitoring parameters increases, the implementation of all the interface circuits into a single headstage becomes more difficult.

An alternative method is to design a headstage with customized ASIC (Mollazadeh et al., 2009; Roham et al., 2009). This allows for the combination of the interface electronics to be incorporated into a single IC, resulting in a smaller headstage dimension and better power efficiency. However, ASICs are designed for specific application and have limited configurability or upgradability. Furthermore, compared to COTS ICs, ASICs require much higher development cost and longer development time. Consequently, ASICs are not suitable at the early research stage. ASICs are more appropriate at a later stage of system design, when all the required system specifications are better understood and verified. Therefore, in a brain research laboratory, the system with headstage built from COTS ICs is preferred as it has lower cost, faster development time and higher configurability.

In this paper, we describe a method for realizing multimodal brain monitoring in freely moving rats by using a cost and time effective headstage. To demonstrate this, we designed a headstage for our previously described multimodal neural probe, which is capable of sensing multiple physiological, biochemical, and electrophysiological parameters simultaneously (Li et al., 2009, 2012, 2015, 2016a,b; Li and Narayan, 2014). This headstage has allowed us to transcend multimodal brain monitoring from anesthetized animals (Li et al., 2016a) to freely moving animals. It provides a reliable electrical/mechanical connection between multiple sensors and their signal processing systems. It also contains essential circuits to improve recording quality for low-level brain signals. The high-level or less noise sensitive parameters are processed at a remote system in order to reduce the design burden (i.e., dimension and weight) on the headstage. The headstage is tethered to a remote signal processing system and power supply through a commutator. While the movement of animal is somewhat restricted in a tethered system, the design requirements for the headstage are more flexible in terms of power consumption and size.

Our system was validated in freely moving rats by simultaneously measuring four cerebral parameters: direct-current electrocorticography (DC-ECoG), brain oxygen tension (PbrO_2_), cortical temperature, and regional cerebral blood flow (rCBF). The developed headstage is user-configurable and allows fast realization of multimodal brain monitoring in freely moving animals. This system has tremendous potential in not only investigating new hypothesis in brain research, but also for verifying system requirements and specifications of newly developed neural probes.

## Materials and methods

### Overview

**Figure 1:**
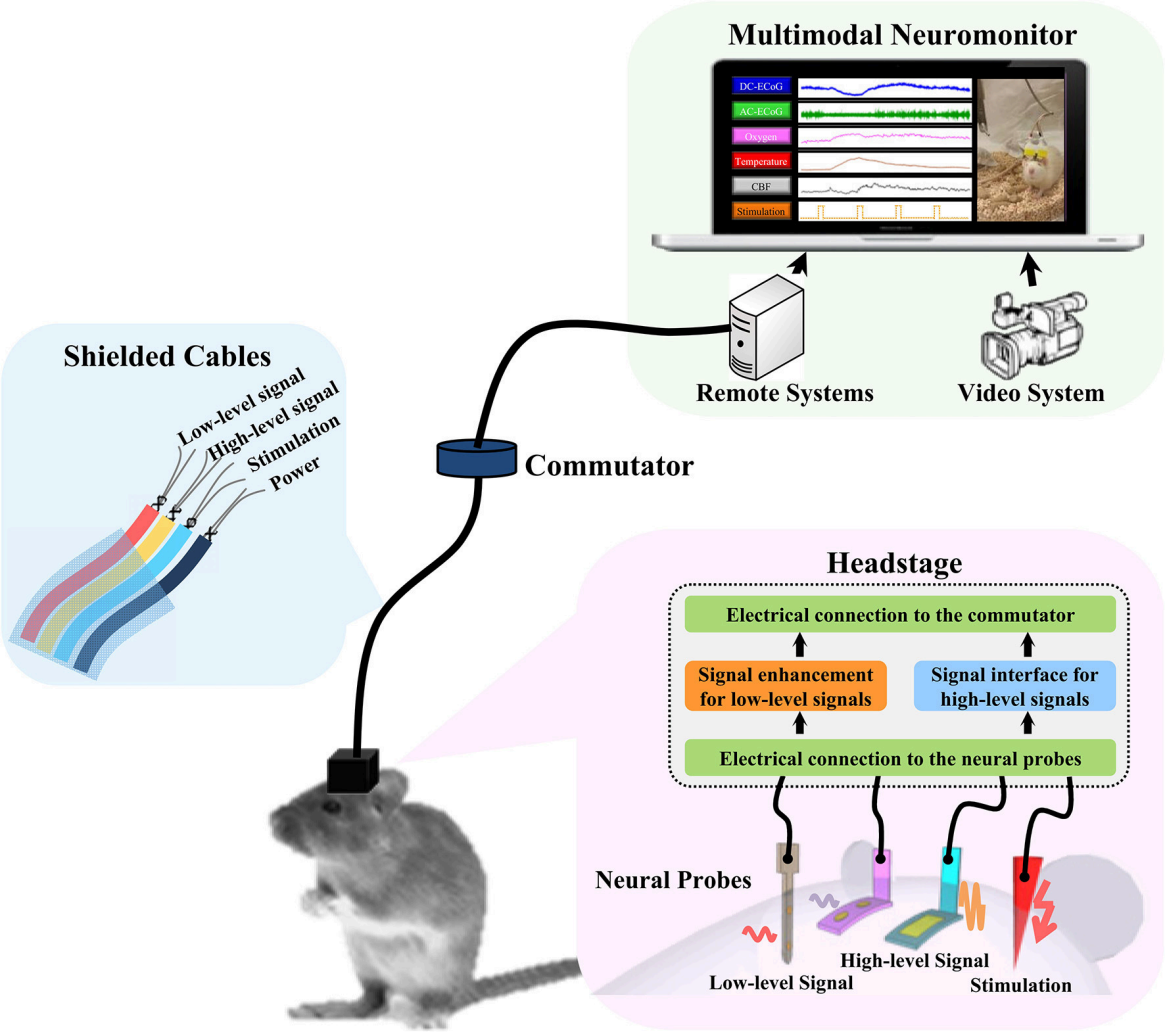
**The concept diagram of multimodality neuromonitoring system in freely moving rats**. The system consists of multiple neural probes, a user-configurable headstage, shielded cable bundles, a commutator, and remote signal processing systems. The headstage amplifies the low-level brain signals and provides the electrical interfaces to the high-level brain signals being processed remotely.

The design concept of the headstage prototype is shown in Figure 1. The system consists of a headstage, shielded cables, a commutator, and remote signal processing systems. The headstage is the key component to realize the multimodality monitoring in freely-moving rats. It serves as both an electrical and a mechanical bridge to signal processing systems. Brain signals can be divided into low-level and high-level signals. In general, low-level signals are more prone to environmental noise. In order to minimize the weight and the dimensions of the system, only the low-level signals are amplified at the headstage. The high-level signals are processed remotely to reduce the complexity of the headstage design. The headstage is linked to the remote system with a shielded cable through the swivel commutator which allows the rodents to move freely without getting tangled. To avoid the signal crosstalk and electromagnetic interference (EMI), the wires inside the cable are properly grouped and shielded between different sensor channels (Ott, 1988). The remote system is used for signal processing and display. It may include interface circuits for high-level signals, synchronized video system, brain stimulator, etc.

### The headstage and interface circuits

**Figure 2:**
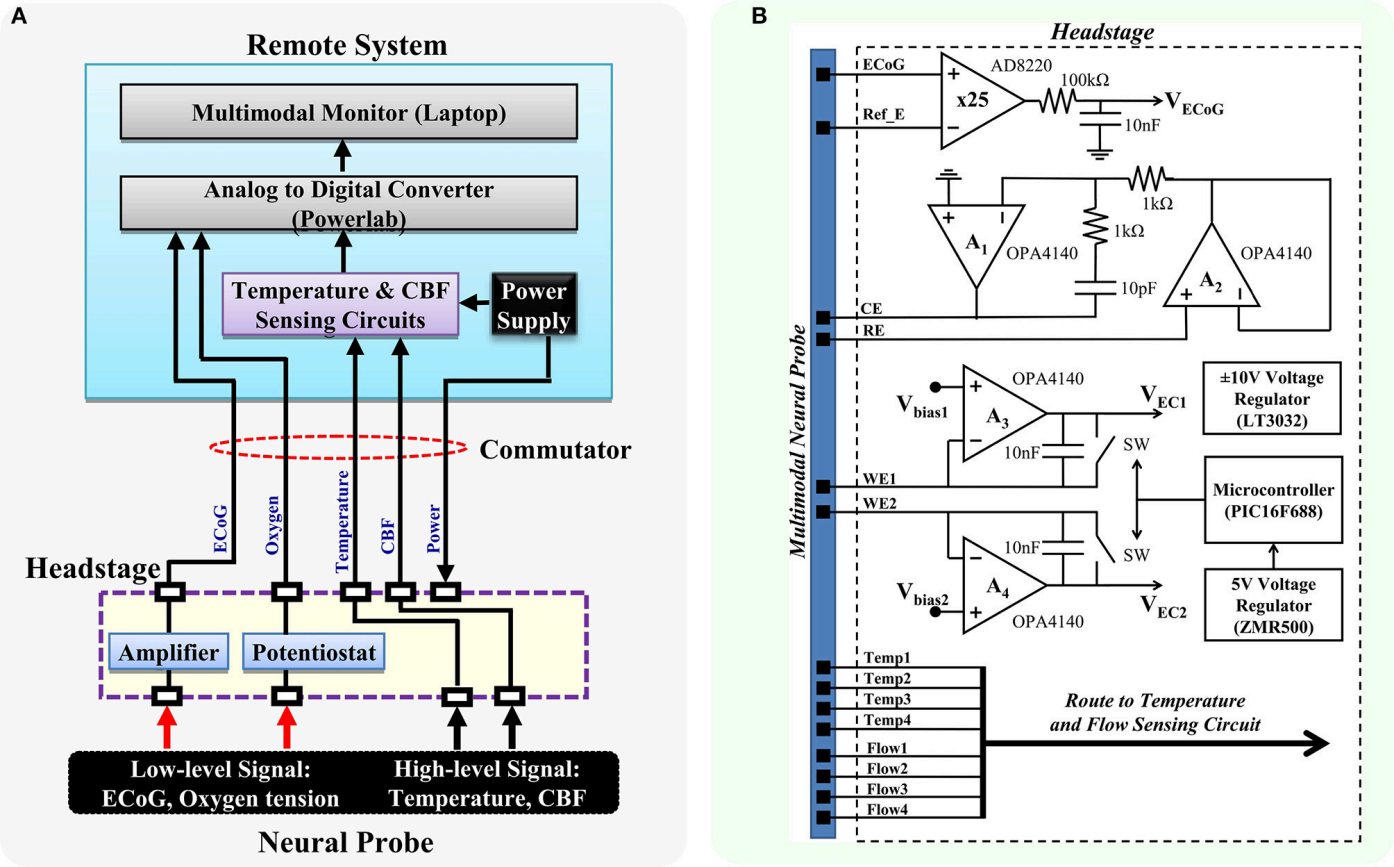
**Block diagram of the proposed multimodality neuromonitoring system. (A)** The proposed system is capable of simultaneously recording ECoG, brain oxygen tension, cortical temperature, and cerebral blood flow in awake tethered rats. **(B)** Detailed diagram of the interface circuits in the headstage. It contains circuits for 1-ch ECoG and 2-ch potentiostat, and electrical interfaces for high-level signals including cortical temperature and cerebral blood flow. RE, reference electrode; WE1, working electrode channel 1; WE2, working electrode channel 2; CE, counter electrode.

Following the design concept, the headstage prototype was developed to interface with the multimodal neural probe, which is capable of simultaneously recording physiological, biochemical, and electrophysiological variables. The multimodal neural probe composed of microsensors for ECoG, PbrO_2_, temperature, and rCBF measurement (Li et al., 2016a). The ECoG recording electrode with on-probe reference electrode was optimized for DC-ECoG recording to have a very low polarization rate and stable baseline for detecting the DC shift of spreading depolarization (Li et al., 2016a,b). The PbrO_2_ microsensors are electrochemical microsensors with 3 electrodes configuration (Li et al., 2016a). The temperature sensor is a resistance temperature detectors (RTD)-based microsensor with four-wire configuration. The rCBF is sensed by the thermal diffusion flowmetry-based measurement technique. The block diagram of the headstage is shown in Figure 2A. The signals from the neural probe are divided into low-level and high-level signals. The headstage contains the interface circuits for low-level signals, which include ECoG (1-ch) and PbrO_2_ microsensors (2-ch). It also provides the electrical contacts to the remote interface circuits for high-level signals, i.e., temperature (1-ch) and CBF (1-ch).

Figure 2B shows the schematic diagram. The ECoG sensing circuit is an instrumentation amplifier (AD8220) with the gain of 25. A first-order RC low-pass filter with the cutoff frequency of 160 Hz is added to the end of the ECoG output to further reduce the noise. The overall input-referred noise from the ECoG circuit is ~0.5 μVrms (grounded inputs). The 2-ch potentiostatic circuit is optimized for measuring brain oxygen tension. The digital control circuit on the headstage includes a 5 V voltage regulator and a small microcontroller. It is used for controlling the operation of the headstage switches and the sampling rate of the potentiostat circuit. The potentiostat has a sampling rate of 5 Hz and a resolution of 0.12 nA in the operation range of 0–0.95 μA, which corresponds to an oxygen tension up to 250 mmHg. Since the power supply of the headstage is provided remotely through a commutator, a ±10 V voltage regulator (LT3032) is used for converting the ±12 V external supply to ±10 V low noise power supply, which provides ample voltage headroom for DC-ECoG recording. The headstage consumes power of ~209.4 mW.

The previously reported temperature and CBF sensing unit (Li et al., 2012, 2016a) has complex circuit components which results in a large interface circuit area (9.7 × 6.4 cm). Therefore, it is placed remotely and connected to the headstage through the swivel commutator. A pulsed four-wire ratiometric measurement method is used for the temperature and flow sensing units to achieve a high sensing accuracy. This also reduces the thermoelectric effects from the electrical contacts of the connectors and the swivel commutator. The temperature sensing circuit has an operation range of 0–2.5 V corresponding to the temperature range of 26.3–51.3°C. It has programmable sampling rate of 10 Hz, and resolution of 1.5 mV. The signal conditioning circuit for the flow sensor has an operation range of 0–2.29 V, which corresponds to an absolute flow range up to 180 ml/100g/min. It has a resolution of 0.7 mV. The power consumption of the temperature and CBF sensing circuit is ~484 mW with ±12 V supply. The configuration and electrical properties of the developed system are shown in Table 1. The electrochemical current and temperature operation ranges were calculated based on the minimum and maximum possible circuit output levels. The dummy 100-Ω resistor was connected to the CBF circuit to measure the maximum CBF operation range. The system resolution was measured by submerging multimodal neural probe in phosphate-buffered saline (PBS) and all parameters resolution was observed after digitization by LabChart software (ADInstruments Inc.).

**Table 1:** **The specification of the developed multimodality neuromonitoring system**.

**Properties**	**Value**
Power supply	±12 V
ECoG gain	25
ECoG noise (grounded inputs)	0.5 μVrms
Electrochemical current range	0–0.95 μA
Current resolution	0.12 nA
Temperature range	0–2.5 V
Temperature resolution	1.5 mV
CBF range	0–2.29 V
CBF resolution	0.7 mV
Headstage power consumption	209.4 mW
Temperature and CBF circuits power consumption	484 mW

**Figure 3:**
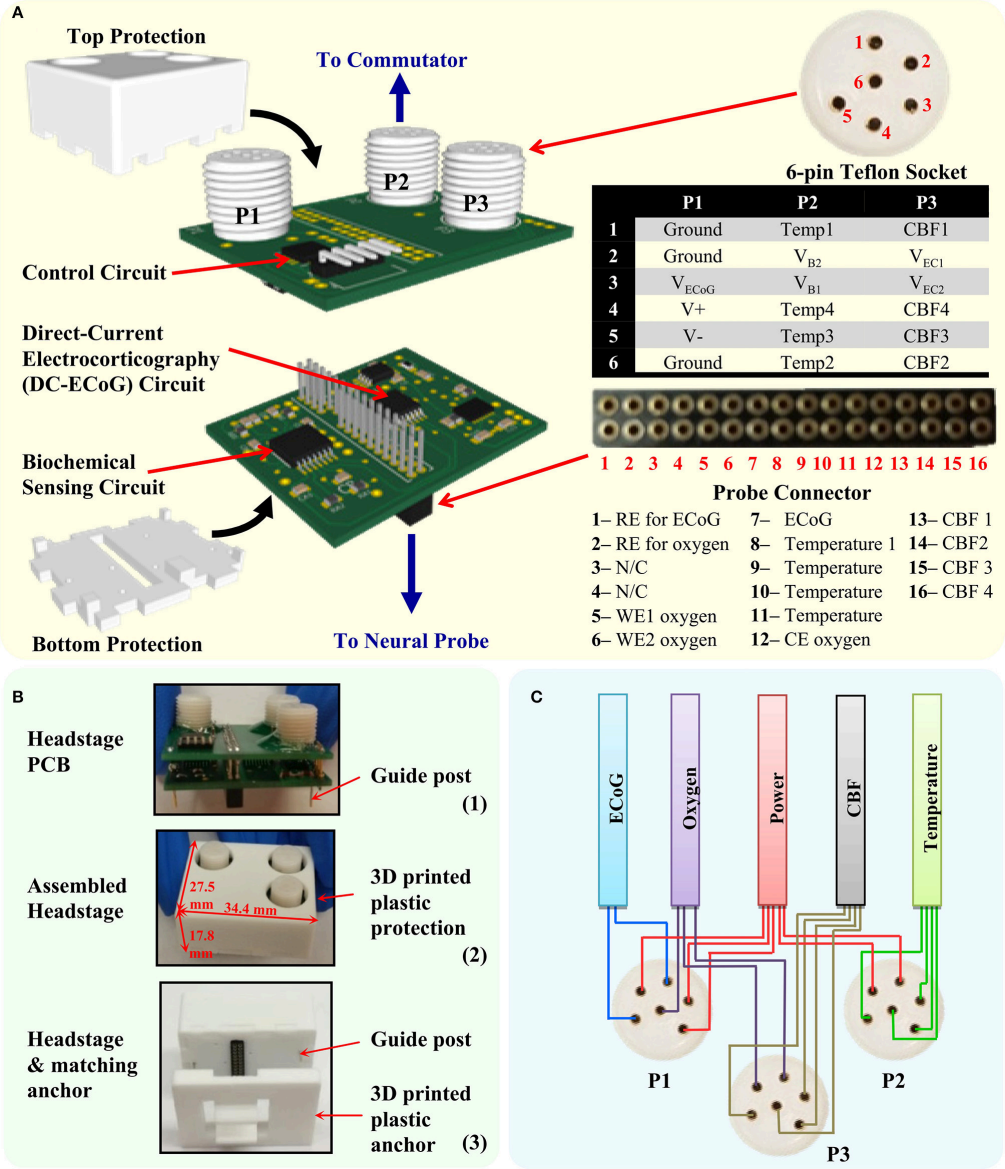
**The developed headstage. (A)** The headstage PCB design consists of two PCBs and a 3D-printed protection case. The bottom level PCB contains the circuit for DC-ECoG and biochemical sensing. The top level PCB contains the headstage control circuit. The headstage is connected to the neural probe through the multi-pin connector and to the commutator through three 6-pin Teflon socket. **(B)** Photography of the headstage PCBs, assembled headstage and the headstage with matching anchor. **(C)** Cable wiring diagram from the Teflon sockets (P1–P3) to avoid crosstalk between sensors.

The headstage design is shown in Figure 3A. It consists of two print circuit boards (PCB) and a 3D-printed electrical/mechanical protection case. The first level PCB is used for signal enhancement for low-level signals (1-ch ECoG and 2-ch oxygen sensors) and interfaces with the multimodal neural probe. The assembled 32-pin connector (Millmax, 853) realizes the electrical connection to the implanted neural probe and provides electrical connection between the two PCBs. The second level PCB houses the digital control circuit and three 6-pin Teflon sockets (PlasticsOne, MS363 with E363/0). The socket-collar plug connections enable secure connections between the headstage and the 18-channel commutator. In order to reduce the number of commutator channels, the digital control circuit is placed in the headstage PCB instead of the remote interface circuit as in the previous design (Li et al., 2016a). Furthermore, to minimize the dimension and weight, COTS ICs with smallest available packages were chosen for the implementation. The 3D-printed protection case was developed to protect the PCBs from mechanical and water damage, and also to minimize movement artifacts.

Figure 3B shows the photography of the developed headstage PCBs, assembled headstage with protection case and its matching anchor. The fully assembled headstage has the dimension of 34.4 × 27.5 × 17.8 mm and weight of 9.6 g. The customized 3D-printed anchor is made with plastic and designed to match the bottom of the headstage. It is used for supporting the neural probe's multi-pin connector. The guideposts under the headstage are designed to tightly match with the holes in the anchor in order to mechanically lock the headstage with the anchor. It also helps to achieve the correct connection polarity between the headstage and the neural probe. To reduce EMI effects, the assembled headstage is wrapped with grounded shielding tape (Laird Technologies, 86750). Table 2 shows the properties of the developed headstage.

**Table 2:** **Properties of the developed headstage**.

**Properties**	**Value**
DC-ECoG	1 channel
Brain oxygen tension (PbrO_2_)	2 channels
Cortical temperature	1 channel
Cerebral blood flow (CBF)	1 channel
Headstage dimensions	34.4 × 27.5 × 17.8 mm
Assembled PCB weight	6.8 g
Protection case weight	2.8 g
Cost	<$150
Development time	<2 weeks

### Signal cabling

In the multimodal brain monitoring system, different types of signals are conducted through the tethered cable including amplified analog voltage outputs from ECoG channel, reset-and-integrate measurement voltage outputs from oxygen channels, current, and voltage pulses from the temperature and CBF channels and the DC power supply. The signals from oxygen, temperature and CBF sensing cables, and DC power supply can generate electromagnetic wave which causes crosstalk noise in the nearby conductor. Such noise can significantly degrade signal quality, especially the amplified analog ECoG signal. Moreover, the signals are susceptible to the EMI radiated from other sources such as AC power and other lab equipment.

The 18-channel cable (Plastics One, 363/3-363/3) is normally used with the 18-channel commutator (Plastics One, SL18C) in the tethered system. While it is good to use a single cable for multichannel recordings of the same parameter such as ECoG, it is not suitable in multimodality monitoring, where the parameters measured can be a combination of high and low level signals. To achieve low noise recording in all the sensing channels and maintain its flexibility, a tether cable bundle is customized for the multimodality monitoring system. The signals from each type of sensors are connected by separate cables to increase their physical separation. This decreases the possibility of signal crosstalk. The cable bundle is composed of five subminiature ultra-flexible instrumentation cables (Brim electronics, 1211/5) for headstage power supply, ECoG, PbrO_2_, temperature, and CBF channels, respectively. Both ends of the cable are soldered to the 6-channel plugs with captive collar (Plastics One, 363/CP) to insure a good electrical and mechanical connection to the headstage and the commutator. The five cables are tied together to form a single flexible cable bundle. Figure 3C shows the connection diagram of the cables to the headstage. To provide better noise immunity to the ECoG channel, its cable shield is connected to the ground.

### Implantation of neural probes and anchors

#### Surgical procedures

**Figure 4:**
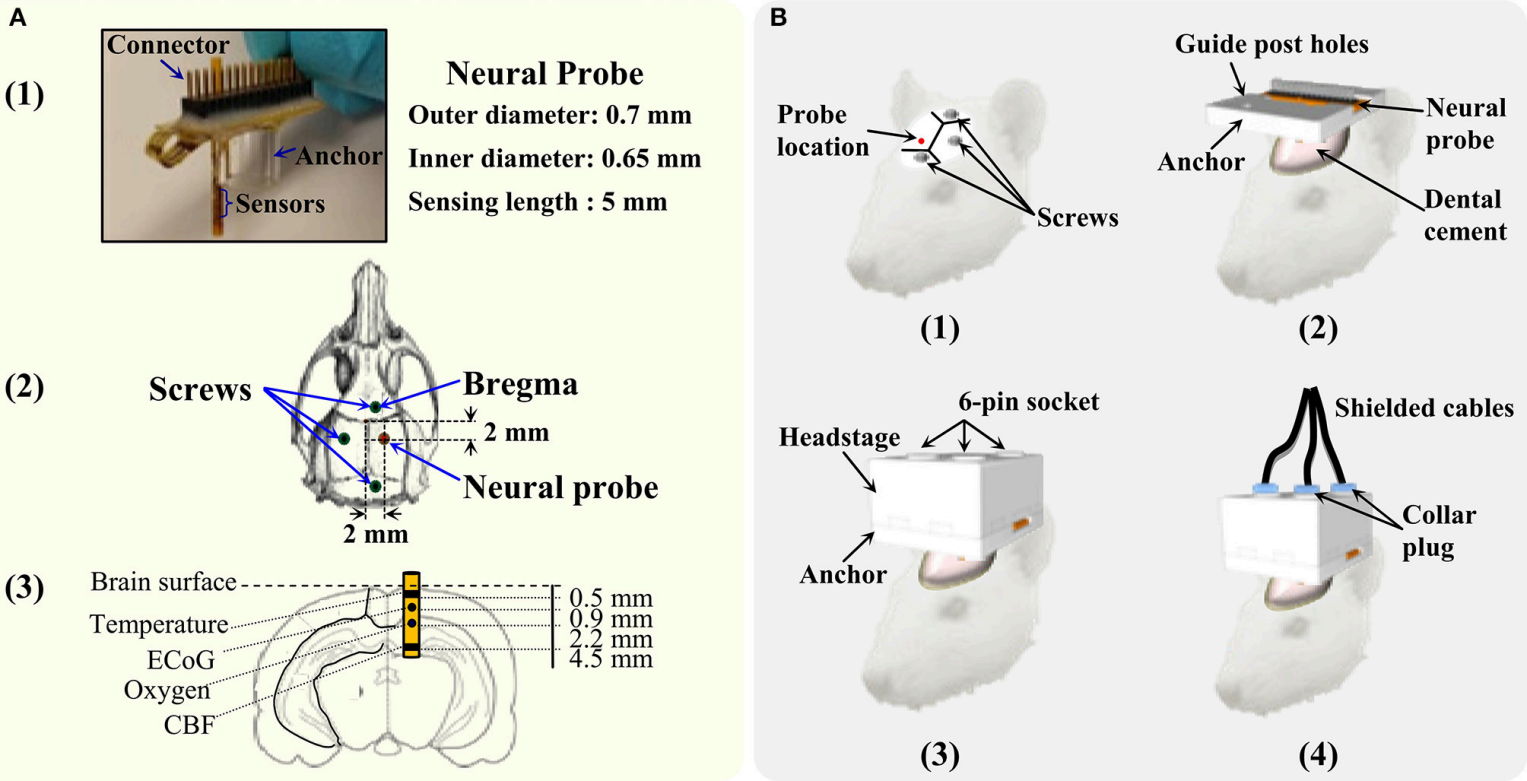
**The details of the multimodality neural probe and surgery procedure of implantation. (A)** Photography of the micromachined multimodality neural probe and coordinates of implantation. **(B)** The steps for the neural probe implantation and connecting the headstage and its cables for data recording.

Male Sprague Dawley rats, weighing 350–450 g, were used in these experiments. For surgical procedures, anesthesia was induced with 5% isoflurane delivered in medical air and maintained at 2.5% throughout surgery. Body temperature was maintained at 37.0°C by a heating blanket. All surgical procedures were approved by the Feinstein Institute for Medical Research Institutional Animal Care and Use Committee. Four rats were used to evaluate the developed tethered multimodal monitoring system. The rats were anesthetized and placed in a stereotaxic frame (David Kopf Instruments, USA). Figure 4A shows the picture of the multimodal neural probe and the diagram depicting approximate placement of neural probe and each microsensor. Microsensors that measure cortical temperature, DC-ECoG, brain oxygen tension, and cerebral blood flow were integrated in the single neural probe (inner diameter = 0.65 mm; outer diameter = 0.7 mm). First, three bone anchor screws were placed in the left parietal, frontal and occipital bone. A craniotomy was then made over right parietal cortex (~0.8 mm^2^; ML: +2 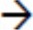 +3 mm; AP: −2 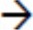 −3 mm; DV: −5.0 mm). The multimodal neural probe was implanted with supporting anchor resting on the skull. The neural probe with the anchor and the screws were cemented into place using dental cement. After 24 h of neural probe implantation, the rats were re-anesthetized (5% isoflurane), and the headstage was connected. Then, the shielded cables were connected to the headstage by locking the collar plugs. Figure 4B shows the steps involved in the neural probe implantation and the headstage connection.

#### Experimental conditions

Animals were provided 25 g of rat feed per day and had water access *ad libitum* before the surgery. Afterwards, both food and water were available *ad libitum*. Animals were individually housed under a 12 h light/dark cycle. On the day of the neural probe implantation, rats were transferred from their cages to custom designed Plexiglas recording chambers (circular cage, Pinnacle Technology Inc.) equipped with multichannel swivel commutators (Plastics One, SL18C). Immediately after surgery, animals were returned to recording chambers. After 24 h following the neural probe implantation, the headstage with shielded cables were connected to the neural probe, and continuous digital records were obtained for the next 72 h.

To increase neural activity and motion, physiological stimulation in the form of a tail pinch was administered. This was achieved by placing a paper clip 3 cm from the tip of the rat's tail for 5 min. This produces a well characterized behavior pattern which consists of gnawing, licking, eating and an overall increase in the level of motor activity (Bolger and Lowry, 2005). At the end of experiment, the rats were deeply anesthetized and euthanized by decapitation.

#### Data acquisition and statistical analysis

Data acquisition was performed using a PowerLab interface system (ADInstruments Inc.). The software package used was LabChart for Windows. Data were reported as means ± SD. To calculate the fluctuation range for each parameter, maximum values were subtracted by the minimum values. Analysis of variance (ANOVA with Newman–Keuls post-test) was used for calculating statistical significance of these changes as a result of physiological stimulation. Comparisons were significant when *p* ≤ 0.05.

## Results

### Signal crosstalk

The signal crosstalk between high-level and low-level signals was evaluated *in vitro* through different tethered cables. In the multimodality monitoring system, the signal characteristic for different sensors can be significantly different. For example, the neural activity in ECoG channel has the level of a few mV after amplification, and the temperature sensor passes 200 μA current with 10 s of mV voltage pulses. The signal running in the temperature sensor wires may be coupled into the ECoG channel. If the coupling is large, it can create a crosstalk and significantly affect the low-level neural activity signal.

**Figure 5:**
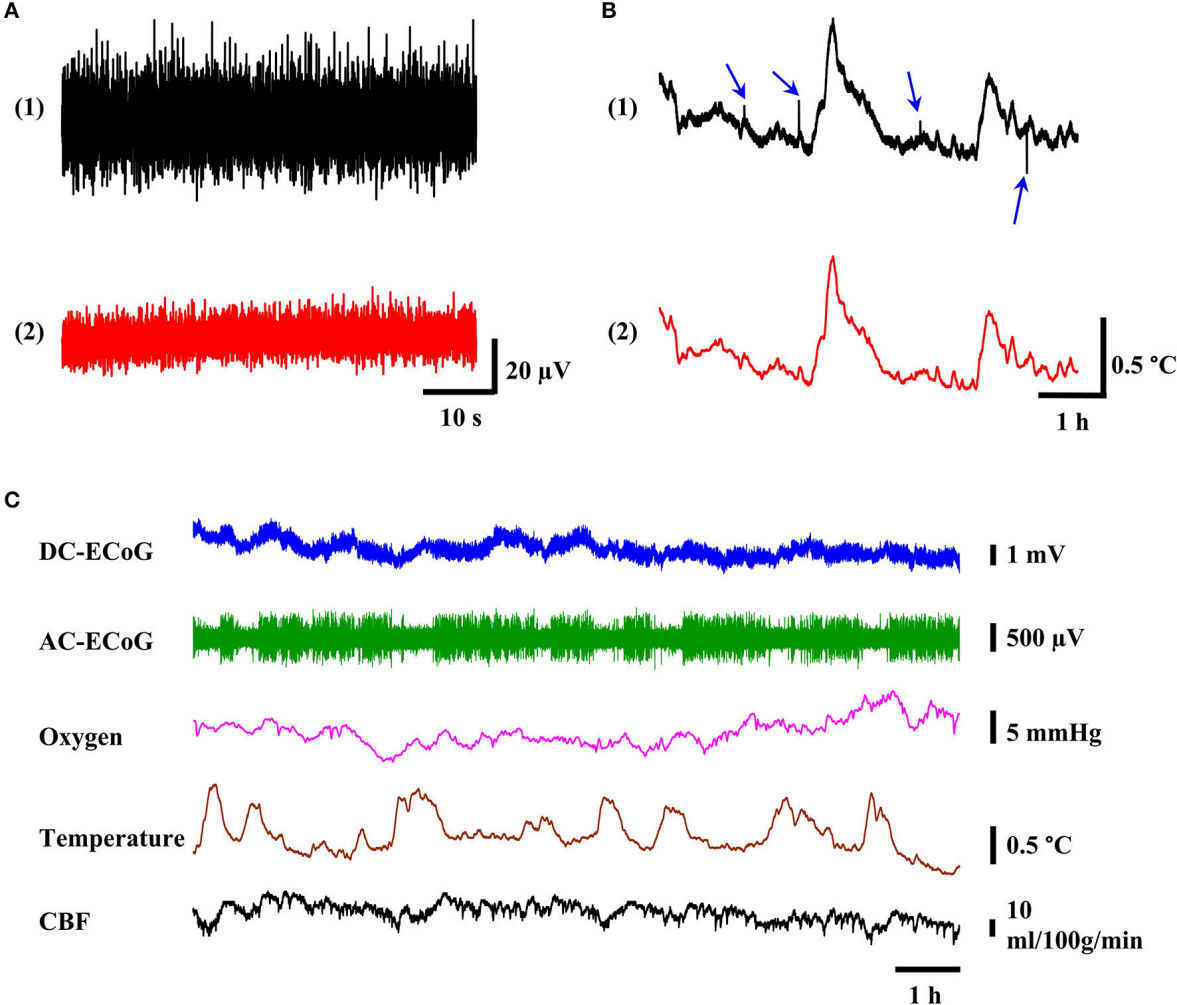
**The quality of recorded multimodality brain signal. (A)** The noise level of ECoG signal before and after physical isolation of cables. **(B)** Representative tracing of 6-h temperature recording (black trace) with noise spikes (blue arrows) and after lowpass filtering with 0.5 Hz cutoff frequency (red trace). **(C)** Example of 12-h reliable multimodality recording of ECoG, oxygen tension, temperature, and CBF signals without significant signal drift and noise spikes.

To avoid the signal crosstalk, the low-level signal channels such as ECoG should be physically separated or provided with proper shielding from the noisy channels (Ott, 1988). However, shielding is difficult to achieve in the tethered system since the commutator does not have the designed channels for the shielding purpose. Hence, in the headstage prototype, the components are physically separated to minimize the potential signal crosstalk. The benchtop test was conducted by using the developed headstage with different cable configurations (18-channel connector cable (PlasticsOne, 363/3-363/3) and customized cable). Unlike the customized cable, all the signal wires were in a single cable for the 18-channel connector cable. The neural probe was submerged in PBS solution. The noise in the ECoG channel after 0.5–50 Hz filtering (i.e., alternating-current ECoG or AC-ECoG) was measured (Figure 5A). The noise with 18-channel connector cable was ~52 μV_p−p_ [Figure 5A (1)]. However, it was reduced to ~25 μV_p−p_ [Figure 5A (2)] with the customized cables. In the customized cables, the cable for P1 (see Figure 3) containing ECoG and power wires was separated from other sensing signals.

### Movement artifact

Movement artifact is a common type of signal contamination in the tethered systems. In the tethered recording setup, noise may come from the movement of the suspended cables through the environment and from the mechanical stresses transmitted to the headstage connectors. In order to minimize the noise from movement artifact, we used multiple screws, dental cement, supporting anchor, and high quality connectors. In 24 h of continuous recordings, we didn't observe any movement artifact in ECoG, brain oxygen tension, and CBF channels. However, we occasionally observed noise spikes in temperature sensor channels. Figure 5B (1) shows one example of such a situation. In a 6-h span of temperature recording, we observed 4 noise spikes (blue arrows). We suspected such noises were from other electrical appliances and equipment such as laboratory refrigerator generating EMI causing interference current in the tether lead (Steenland and McNaugton, 2015). The excitation current for the temperature sensor may be disturbed by the EMI, resulting in noise spikes in the temperature outputs. The reason that we didn't observe similar noise in the ECoG and oxygen channels is most likely because they are amplified and therefore, less susceptible to the noise. In the case of CBF signal, it may be because it has at least 25-fold higher signal levels than the temperature sensor. However, such noises can be removed by using lowpass filter with 0.5 Hz cutoff frequency as shown in Figure 5B (2).

Cortex temperature, DC-ECoG, brain oxygen tension, and CBF were measured simultaneously in freely moving rats for 24 h. The full-band DC-ECoG signal (0.0–160 Hz) was filtered in real-time with LabChart software to display the AC-ECoG signals (0.5–50 Hz) simultaneously. Baseline cerebral physiology was monitored for 24 h with no experimental manipulations to evaluate the system's reliability. Figure 5C shows a representative 12 h plot of these recordings. Twenty-four hours after probe implantation, the baseline values for DC-ECoG, cortical temperature, brain oxygen tension and CBF were 10.3 ± 7.2 mV, 37.4 ± 0.5°C; 27.2 ± 6.9 mmHg, and 42 ± 16 ml/100g/min (*n* = 4), respectively. DC-ECoG experienced 18.2 ± 12.8 mV (*n* = 4) drift during the 24-h continuous recordings. The fluctuation ranges for cortical temperature, brain oxygen tension, and cerebral blood flow were 1.29 ± 0.19°C (*n* = 4), 10.4 ± 7.6 mmHg (*n* = 4); 14 ± 9 ml/100g/min (*n* = 4), respectively for the 24-h recordings. In all the measurements, no movement artifact was observed.

### Multimodal brain monitoring

#### Response to light-dark cycle

**Figure 6:**
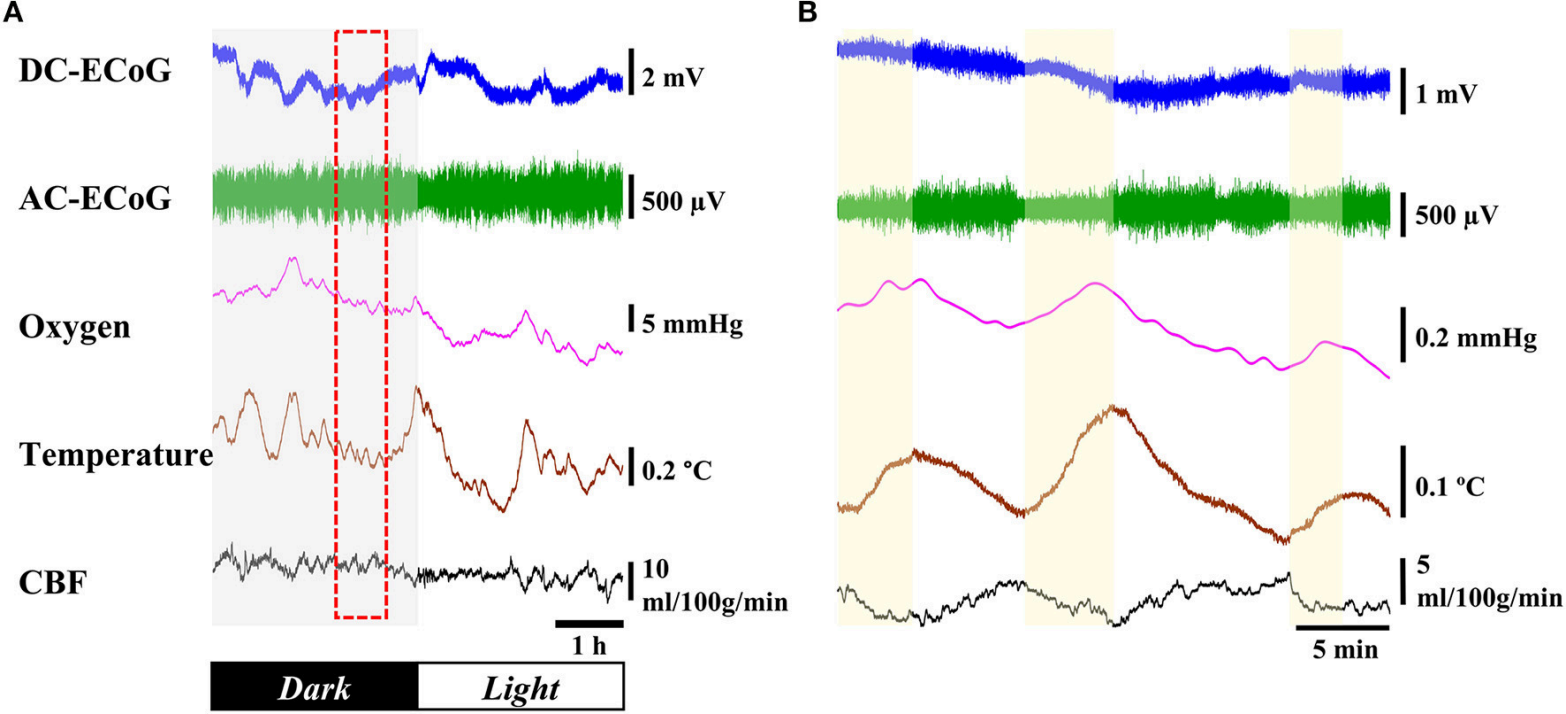
**Response to light-dark cycle. (A)** Example of 6-h recording of the ECoG, oxygen tension, cortical temperature, and CBF through the dark/light cycle. **(B)** Expanded view of dashed box.

Figure 6A shows 6 h data across the dark-light cycle transition. There were no substantial changes for DC-ECoG potential or CBF levels between dark and light cycles. However, during the light cycle, AC-ECoG showed a predominance of high-amplitude synchronous neural activity. In addition, cortical temperature decreased by 0.6 ± 0.1°C (*n* = 4) during the light compared to the dark cycle. Brain oxygen tension progressively decreased by 9.5 ± 1.8 mmHg (*n* = 4) during the light cycle. Figure 6B shows an expanded view of a 30-min data recording during the light cycle (dashed box in Figure 6A). When the amplitude of the AC-ECoG decreased, both cortical temperature and brain oxygen tension increased. The CBF responded to the different trend from cortex temperature during the same response period.

#### Effect of neuronal activation

**Figure 7:**
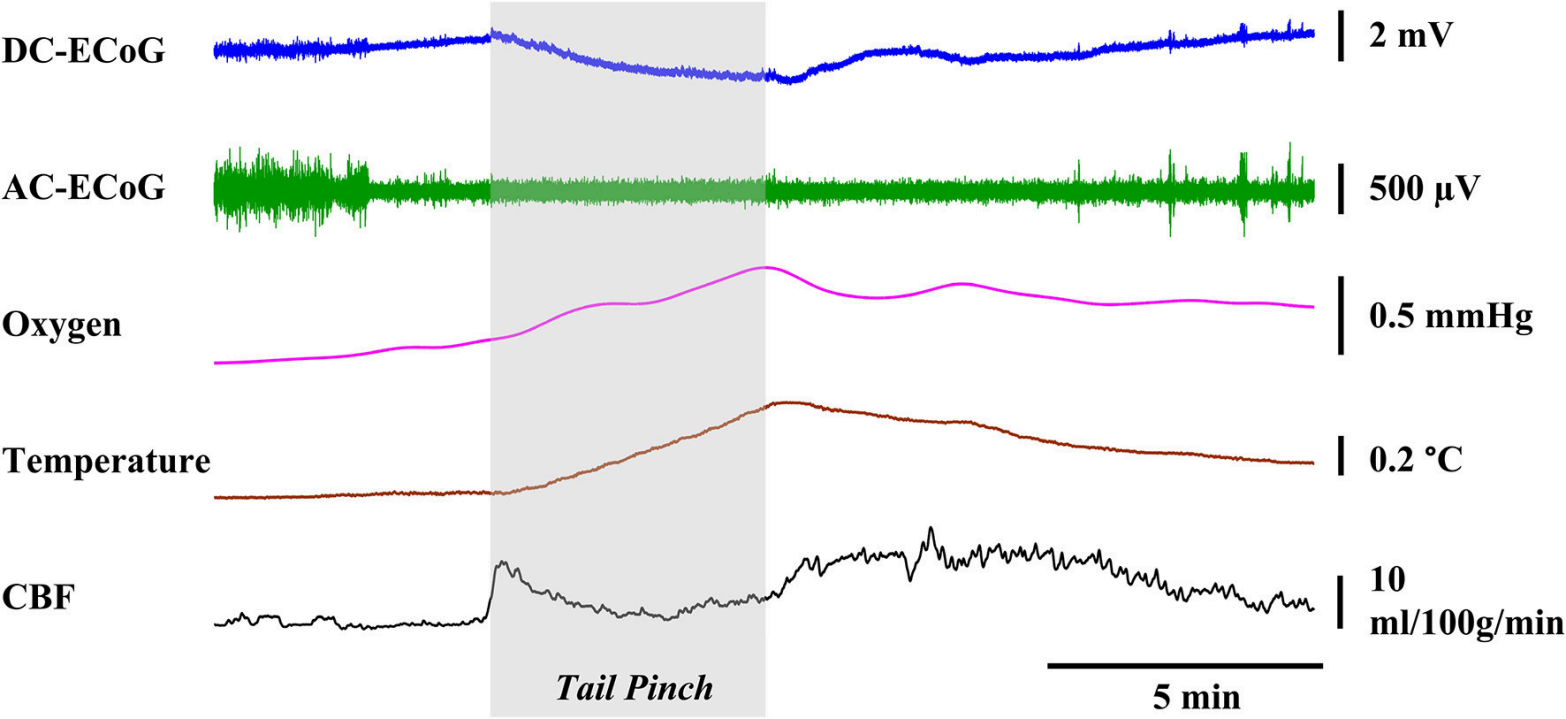
**Representative graphs showing the effects physiological stimulation**. A 5-min tail pinch applied to the freely moving rats resulted in significant changes in the DC-ECoG, cortical temperature, brain oxygen tension, and CBF.

A 5-min tail pinch applied to the freely moving rats (*n* = 4) resulted in DC-ECoG, temperature, brain oxygen tension and CBF changes as shown in Figure 7. Immediately after the stimulus was applied, negative shifts in DC potential occurred (0.84 ± 0.25 mV; *p* < 0.05 vs. baseline). A few seconds after the paper clip application, we observed an enhancement in chewing activities which resulted in a significant increase in brain temperature (0.31 ± 0.03°C; *p* < 0.05 vs. baseline), oxygen tension (4.15 ± 0.68 mmHg; *p* < 0.05 vs. baseline) and CBF (9.15 ± 5.04 ml/100g/min; *p* < 0.05 vs. baseline).

## Discussion

In this paper, we provide a cost and time effective approach to designing a headstage for conducting multimodal brain monitoring in freely moving animals. The developed headstage can reliably record electrophysiological, biochemical, and physiological signals simultaneously, without significant signal crosstalk or movement artifacts for 72 h. It can record low-level neural signals with high quality, and easily interface with signal conditioning circuits that have high power consumption and are difficult to miniaturize. The usage of ±12 V supply for our current system (Li et al., 2016a) allows us to observe signals with wider ranges. However, it leads to high power consumption at ~209 and ~484 mW for the headstage and remote temperature/CBF sensing circuits, respectively. If we use small batteries (e.g., Duracell, two MN21 12V batteries with 33 mAh) for operating the headstage, the recordings would only run for ~3 h. Such short battery life is not suitable for our purpose since we need to perform long-term brain monitoring. Therefore, our system is powered through tethered cables, and provides unlimited hours of monitoring. Moreover, the interface circuit for temperature and CBF sensing (Li et al., 2016a) is too complex to be sufficiently miniaturized using COTS. Therefore, the tethered system approach is preferred rather than the wireless system, especially at an early development stage.

Many tethered or wireless systems have been developed for simultaneous measurement of multiple brain parameters. For instance, multiple neurochemicals such as hippocampal oxygen and glucose were measured in freely moving rats (Kealy et al., 2013). Kang et al. reported continuous telemetry monitoring of multiple physiological parameters (intracranial pressure and brain temperature) in rats (Kang et al., 2016). Rocchitta et al. demonstrated a telemetry system for simultaneous detection of extracellular brain glucose and lactate and motion (Rocchitta et al., 2013), which was the combination of neurochemical and physiological signals. An approach for simultaneous measurement of neurochemical and electrophysiological parameters has also been reported. Dash et al reported long term (2–3 days) simultaneous measurement of brain glucose and electrophysiology in tethered animals (Dash et al., 2013). However, none of these systems offered a configurable platform that allowed us to add different monitoring parameters in a convenient manner. In this paper, we have shown a method to monitor neurochemical, electrophysiological, and physiological parameters in freely moving rats through a user-configurable headstage.

Multimodality brain monitoring experiments confirmed the reliability of the developed system. During the 72-h span of continuous measurement, we didn't observe any signal crosstalk between the sensing channels or any movement artifacts. In the present study, we recorded similar responses of cerebral variables when compared to previously reported sleep-wake cycle signal pattern (Lapray et al., 2008; Kiyatkin et al., 2013) and behavior induced changes in neural activity using tail pinch stimulation. Compared to the awake period, the AC-ECoG signals in the sleep period displayed higher amplitude and lower frequency synchronous discharges; the brain temperature was also lower. Moreover, the fluctuations between synchronized and desynchronized brain states also corresponded to the changes in brain tissue oxygenation (Dash et al., 2013). These changes were observed in all animals (*n* = 4). Little is known about the factors that regulate CBF during sleep-wake cycles. We therefore cannot find a good reference to explain the data we observed. For the tail pinch experiment, we recorded similar changes in brain temperature, oxygen, and CBF (Bolger and Lowry, 2005; Bazzu et al., 2009; Kiyatkin et al., 2013). Physiological stimuli increased neural activity and chewing activities. We observed a significant increase in brain oxygen tension, temperature, and rCBF. Because of the developed headstage, we can now simultaneously record all these responses, which was possible only in part in previous *in vivo* studies.

The headstage for multimodal brain monitoring system is based on COTS components. Hence, it takes advantage of the flexibility in the design for different applications and system requirements, and has a fast turnaround time. The total cost of the headstage including PCBs, components, protection cases is <$150. The development time is <2 weeks by using the COTS and fast PCB fabrication service. The fully assembled headstage weighs 9.6 g and has the dimension of 34.4 × 27.5 × 17.8 mm. It is bulky for smaller animal such as mice with average weight of ~20–30 g (Fan et al., 2011). However, compared to the state-of-the-art existing headstage and wireless solutions (Ball et al., 2014), it is acceptable for rat experiments. In addition, the headstage can be reused many times, which mainly depends on the connector mating cycles. Its parameters are given in Table 2. The future plan for improvement of our design include further reducing the headstage weight and dimension, integrating synchronized video system for correlating the brain parameters with behavioral states (Lapray et al., 2008), and adding the passages for electrical/optogenetic stimulation (Ye et al., 2008; Wu et al., 2013; Pashaie et al., 2014; Pinnell et al., 2015).

The headstage in this study was specially designed for our multimodal neural probe. However, the design concept can be adapted to different neural probes and sensing systems to conduct multimodal brain monitoring in freely moving rats. Rapid prototyping of tools is important for neuroscience research and aids tremendously in designing and conducting experiments. Using our rapid headstage prototyping approach, multimodal brain parameters from single or multiple neural probes can be measured. This can aid in the neurophysiological evaluation of freely moving animals. After all the system functions are verified for a specific application, the optimized interface circuits can be combined into a single ASIC which allows for lighter, smaller, and lower power consumption unit.

The paper describes a unique method for rapid prototyping of a headstage that aids in multimodality neuromonitoring in freely moving animals. From a research perspective, our design is user-friendly and offers flexibility to combine neural probes and other stimulating and imaging devices. It provides a useful adjunct to current brain monitoring systems without significant technology barrier. We anticipate that this tool will play an ever increasing role in helping us gain further insight into both normal cerebral physiology and disease processes such as epilepsy, stroke, and traumatic brain injury.

## Author contributions

KL: designed the method, conducted the experiments, analyzed data, and wrote the manuscript; RN: designed the method, reviewed the manuscript, and supervised the research; AC: wrote the manuscript; EG: analyzed data and wrote the manuscript; CB: wrote the manuscript; CL: conceived and designed the study, conducted the experiments, analyzed data, and wrote manuscript. All authors read and approved the final manuscript.

### Conflict of interest statement

The authors declare that the research was conducted in the absence of any commercial or financial relationships that could be construed as a potential conflict of interest.
